# Assessing the efficacy of asynchronous telehealth-based hearing screening and diagnostic services using automated audiometry in a rural South African school

**DOI:** 10.4102/sajcd.v65i1.582

**Published:** 2018-07-05

**Authors:** Samantha M. Govender, Maurice Mars

**Affiliations:** 1 Department of Telehealth, University of KwaZulu-Natal, South Africa

## Abstract

**Background:**

Asynchronous automated telehealth-based hearing screening and diagnostic testing can be used within the rural school context to identify and confirm hearing loss.

**Objective:**

The aims of the study were to evaluate the efficacy of an asynchronous telehealth-based service delivery model using automated technology for screening and diagnostic testing as well as to describe the prevalence, type and degree of hearing loss.

**Method:**

A comparative within-subject design was used. Frequency distributions, sensitivity, specificity scores as well as the positive and negative predictive values were calculated. Testing was conducted in a non-sound-treated classroom within a school environment on 73 participants (146 ears). The sensitivity and specificity rates were 65.2% and 100%, respectively. Diagnostic accuracy was 91.7% and the negative predictive values (NPV) and positive predictive values (PPV) were 93.8% and 100%, respectively.

**Results:**

Results revealed that 23 ears of 20 participants (16%) presented with hearing loss. Twelve per cent of ears presented with unilateral hearing impairment and 4% with bilateral hearing loss. Mild hearing loss was identified as most prevalent (8% of ears). Eight ears obtained false-negative results and presented with mild low- to mid-frequency hearing loss. The sensitivity rate for the study was low and was attributed to plausible reasons relating to test accuracy, child-related variables and mild low-frequency sensory-neural hearing loss.

**Conclusion:**

The study demonstrates that asynchronous telehealth-based automated hearing testing within the school context can be used to facilitate early identification of hearing loss; however, further research and development into protocol formulation, ongoing device monitoring and facilitator training is required to improve test sensitivity and ensure accuracy of results.

## Introduction

Children comprise 32 million of the 360 million people globally presenting with disabling hearing loss, and it is estimated that almost 9% of hearing-impaired children are 15 years and younger (World Health Organization [WHO], 2017). According to Butler (2012), by using a conservative prevalence of 1% for the combined group, it can be estimated that 1.5 million children under the age of 15 years in South Africa present with some type of hearing impairment. Late diagnosis of childhood hearing loss is associated with lack of early detection and intervention programmes, late identification of hearing loss and prioritisation of life-threatening health conditions over hearing loss (Olusanya, Okolo, & Adeosun, 2004). Developing regions including sub-Saharan Africa, Asia Pacific and South Asia share the greatest burden of hearing impairment (Gell et al., 1992).

Otitis media, wax impaction and hearing loss, particularly mild and unilateral in nature, are the most common auditory pathologies noted in school-aged children (Sarafraz & Ahmadi, 2009). Permanent disabling hearing loss of varying degrees (mild to profound) is also a concern in this population (Yamamah, Mabrouk, Ghorab, Ahmady, & Abdulsalam, 2012). A disabling hearing loss is defined as the pure-tone average (500 Hz, 1000 Hz and 2000 Hz) of 30 dBHL or more in the better ear (WHO, 2012).

Irrespective of the time of onset, cause or severity, undiagnosed or late-diagnosed hearing loss in children can negatively impact on academic performance, social and emotional development as well as vocational opportunities (Janardhana, Muralidhar, Naidu, & Raghevendra, 2015). The emphasis on educating children holistically has drawn focus to the multiple dimensions that influence learning and academic progress (Kochhar-Bryant & Heishman, 2010). It has long been recognised that early identification and timely management of auditory pathology in children translates into positive outcomes (Yoshinaga-Itano, Sedey, Coulter, & Mehl, 1998). However, although school hearing screening programmes are a reality in most developed countries, developing countries still lack such services as a result of resource constraints (Monica et al., 2016). Developing countries such as India, Kenya, Nigeria and South Africa have attempted to develop and implement school-based healthcare services that include hearing screening (Balarajan, Selvaraj, & Subramanian, 2011; Department of Basic Education [DoBE], 2012; Oyinlade, Ogunkunle, & Olanrewaju, 2014). The inclusion of hearing screening is within the healthcare package offered as part of the Integrated School Health Programme (DoBE, 2012) to South African schools, an initiative of the South African government. However, several challenges relating to implementing and sustaining hearing screening services within the school context have been highlighted.

These challenges include lack of diagnostic follow-up and continuation of care (Govender, Latiff, Asmail, Ramsaroop, & Mbhele, 2016; Jin, Sklar, Oh, & Li, 2008), lack of efficient transport systems, financial difficulties, long travel distances and negotiating long waiting queues in public sector hospitals. Furthermore, parents face challenges in obtaining leave of absence from work, thereby adding to the poor follow-up process (Jones, Sherman, & Varga, 2005). Failure of the referral system defeats the primary purpose of hearing screening which is to identify children with hearing loss and enable diagnosis and timely management (Mahomed-Asmail, Swanepoel, & Eikelboom, 2016a; Skarżyński & Piotrowska, 2012). Furthermore, high-noise levels when conducting conventional screening at schools also posed challenges to the screening programme as this can result in high false-positive rates and associated unnecessary referrals (Lo & McPherson, 2013).

Introducing diagnostic hearing testing within the school environment could remedy some of these challenges, such as poor follow-up of recommendations made, as well as unnecessary referrals. However, conventional diagnostic testing in schools requires infrastructure and special considerations including sound-treated facilities and diagnostic audiometers. There are also too few audiologists globally, including in South Africa, to ensure that these services can be adequately and equitably delivered to all children (Swanepoel et al., 2010). The use of automated telehealth applications in audiology within schools may alleviate some of these challenges. Telehealth-based audiology services allow for service delivery to remote and rural areas as such testing can be done outside a sound-treated environment. Noise cancellation headphones allow for testing to be conducted outside the sound-treated room within a noise-controlled environment (Swanepoel, Maclennan-Smith, & Hall, 2013). Such headphones provide an opportunity for both screening and diagnostic services to be delivered at schools, thereby possibly closing the gap between identification and intervention. Automation of audiology equipment allows for standardisation of test procedures and protocols, improvement in diagnostic accuracy by reducing clinician variability, and efficient electronic record keeping (Brennan-Jones, Eikelboom, & Swanepoel, 2017; Mahomed, Swanepoel, Eikelboom, & Soer, 2013; Margolis & Morgan, 2008; Mars, 2013; Storey, Munoz, Nelson, Larsen, & White, 2014).

Applying an asynchronous, store and forward model for both screening and diagnostic assessment allows testing to be performed by a trained assistant with the results subsequently evaluated by an audiologist at a different location and time (Swanepoel et al., 2010). Therefore, a store and forward model provides an opportunity for testing to be done at any time by a trained facilitator, and the results can be stored in a password-protected computer and subsequently sent to a qualified audiologist anywhere in the world for interpretation and analysis. Screening coupled with an asynchronous telehealth diagnostic service delivery model may be beneficial in rural areas in South Africa and other developing countries where access to services is limited. Such a service delivery model optimises human resource distribution in resource-constrained settings.

Existing studies confirm that diagnostic automated testing can yield reliable results (Brennan-Jones et al., 2017; Mahomed-Asmail, Eikelboom, Myburgh, & Hall, 2016b). However, only a few diagnostic studies using automated audiometry have been conducted in schools. In addition, available studies primarily focus on validation as well as comparing conventional results to automated findings. Based on current evidence, asynchronous tele-audiology services using automated technology may be used as an effective service delivery model in schools; however, more empirical evidence is required to assess the efficacy of this model.

A review of the literature shows that telehealth services can be used to deliver audiology services within the school context; however, not all these studies used automated technology. In addition, only a few studies conducted both screening and diagnostic assessments.

Remote hearing screening via telehealth using manual audiometry was conducted at a rural elementary school in Utah in the United States on 32 Grade 3 children (Lancaster, Krumm, Ribera, & Klich, 2008). Each participant received one screening on site and another through telehealth procedures. No statistically significant differences were found between results obtained through remote pure-tone screening and on-site screening. The researchers did find five participants who performed differently during the telehealth method and suggested that more studies were required to understand the reasons for false responses. Diagnostic pure-tone audiometry within two urban school contexts without a sound-treated environment was evaluated (Swanepoel et al., 2013). A total of 149 children were evaluated asynchronously both within a soundproof booth with conventional audiometry and with a computerised device, the KUDUwave 5000. The participants were purposively selected and none presented with significant hearing loss. In addition, any participants presenting with middle ear pathologies during the middle ear assessments were also excluded from the final sample. Both air and bone conduction testing were performed, and no statistically significant differences were found between thresholds recorded under both conditions.

Monica et al. (2016) conducted a synchronous tele-hearing screening service in a school. Remote computing software was used so that the audiologist could remotely operate the audiometer whilst the facilitator set up and instructed the participant at another school. TeamViewer was used to provide videoconferencing and synchronous services between both sites for screening to be conducted. Thirty-one children participated in the study. All participants were screened using telehealth and conventional methods of screening with no statistically significant differences between methods. In the studies conducted by both Swanepoel et al. and Saleth et al., only manual audiometry was utilised which is different from the context of the present study, which aims to evaluate the use of automated audiometry within the natural school environment.

A telehealth model using synchronous audiometry was used to conduct hearing screening on 143 children in a study by Skarżyński et al. (2016). Hearing impairment was identified in 34 participants (23.7%). The study supported the use of a telemedicine model to assess hearing status in children within the school context. This study also used a manual protocol for assessment. Mahomed-Asmail et al. (2016b) investigated the validity of automated diagnostic air and bone conduction audiometry for children in a natural school environment following a hearing screening test. No significant differences were found between manual and automated air and bone conduction audiometry. This study conducted screening with smartphone technology and only used the KUDUwave 5000 for diagnostic testing. The study highlighted some limitations, including that testing was only conducted down to 15 dBHL as that was considered the cut-off for normal hearing, and the authors recommended that future studies conduct testing lower to 0 dBHL. The study also recommended that testing should be done in more natural school environments with adverse acoustic conditions.

The purpose of the present study was to build on the work conducted in automated audiometry by evaluating the efficacy of an asynchronous model using automated audiometry to conduct screening and diagnostic testing. This was considered necessary because most of the studies reviewed have either conducted synchronous testing or used manual audiometry as opposed to automated audiometry.

## Research method and design

### Aims

The aims of the study were to evaluate the efficacy of an asynchronous telehealth-based service delivery model using automated technology for screening and diagnostic testing in a rural school in South Africa as well as to describe the prevalence, type and degree of hearing loss found in the study sample.

### Study design

A comparative within-subject design was used. A within-subject design ensures that every participant receives the test that is under investigation and serves as his or her own control. This reduces the amount of error that may arise when comparing variables between participants (Charness, Gneezy, & Kuhn, 2012). The disadvantage of this design is related to the ‘carry-over effects’, where a participant’s performance in one test may influence his or her performance with the other test and therefore needs careful consideration.

### Study site

The study was undertaken at a rural primary school in the Bojanala District of the North-West province in South Africa.

### Sampling method

The entire population of learners at the school (*n* = 300) was invited to participate and were given invitation letters, along with consent forms, information documents and letters of assent to take home to their parents. Only those learners who returned a signed consent form were included in the study.

### Participant description

Participants were from Grades 1 to 7 and ranged in age from 6 to 12 years with a mean age of 8.9 (SD *=* 1.99). Both genders were adequately represented in the study sample with 60% girls and 40% boys.

### Equipment

The otoscopic examination was conducted using a DE500 Firefly digital video otoscope and various sizes of specula. Screening and diagnostic automated audiometry was conducted using the KUDUwave 5000 type 2 clinical audiometer (eMoyoDotNet, Johannesburg, South Africa). The KUDUwave 5000 was connected to an HP ProBook laptop with a Windows 10 operating system. The KUDUwave provides ‘Ambidome’ technology, which has built-in soundproofing, replacing the soundproof booth (KUDUwave user manual, 2013). It uses a combination of sound dampening and real-time monitoring of ambient noise. The device connects to circumaural earphones that attenuate ambient noise through two microphones positioned on the headphones that monitor the noise levels. Deeply inserted foam ear tips, connected to two detachable sound tubes, connected to the KUDUwave were placed into the ear canal of the participant with the circumaural headphones placed over both ears. A B71 bone oscillator was used for bone conduction testing and was kept in position on the head by a screw-on headband. The bone oscillator was placed on the forehead. The patient was given a response button connected to the KUDUwave, allowing automated recording of the responses. The KUDUwave was a new device and was calibrated by Emoyo.Net a month before use. The GSI 33 middle ear analyser was used to conduct tympanometry testing. Classification of tympanograms was made according to the shape, middle ear pressure, ear canal volume and static compliance in accordance with the study by Jerger (1970).

### Facilitator training

The facilitator was recruited through a local doctor in the Bojanala District who was familiar with some of the community volunteers. She could speak English as well as the local language (Setswana) and was computer literate. The facilitator was trained by the primary investigator (SG) who had been trained by a KUDUwave representative. Facilitator training took place over 2 days. Day one consisted of an overview of the hearing screening protocol and orientation to the KUDUwave and the digital Firefly otoscope. Aspects including proper handling and setting up of the equipment, patient set-up, patient information entry and troubleshooting were addressed. Day two comprised a practical component where the assistant was required to conduct screening of 10 children under the observation of the primary investigator. The primary investigator was also present for the first day of screening to ensure that no problems were experienced by the facilitator. The assistant was also given a copy of the KUDUwave manual (2013:revision 3) for reference.

### Test environment

Testing was conducted in the school library, which was approximately 20 m away from the playground and classrooms. During testing, no children were allowed access to the library. Testing was interrupted during break time as a result of increased noise levels. Noise level measurements were taken intermittently (an average of three readings per day) to ensure that the noise levels were kept below 50 dBSPL. There were only a few occasions when the noise levels reached an average of 60 dBA after the three readings and testing was interrupted during these times.

### Participant preparation

Participants were conditioned for both screening and diagnostic testing before the assessment began. They had the opportunity to ask questions, to listen to practice tones and were orientated to the response button. Instructions were given in a simple manner in the first language of the participant.

### Data collection procedure

#### Screening phase

**FIGURE 1:**
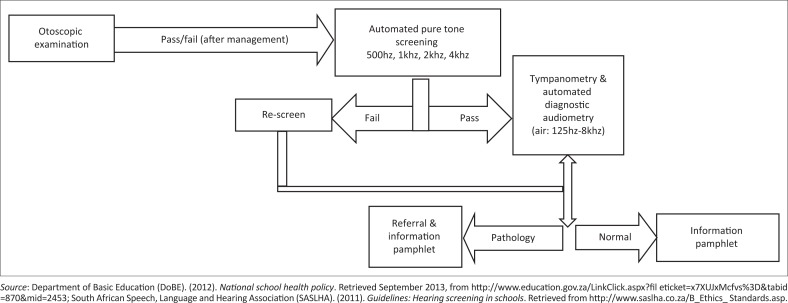
Clinical protocol.

The screening protocol as outlined by SASLHA (2011) was adhered to (Figure 1). An asynchronous telehealth model was applied. A trained facilitator conducted the screening, and the results were asynchronously evaluated later by a qualified audiologist. Participants first underwent an otoscopic examination performed by the facilitator using a DE500 Firefly digital otoscope, which is a laptop-based video otoscope programme. Video recordings of the participants’ external auditory canal and tympanic membrane were saved in a password-protected folder on the computer. These recordings were studied asynchronously by the audiologist daily after the participants were screened. A healthy outer ear and external auditory canal and presence of a light reflex from the tympanic membrane were landmarks for normal otoscopic findings. Participants presenting with pathologies on otoscopy such as impacted cerumen, perforated tympanic membranes or fluid around the tympanic membrane were referred to general practitioners in the area for further evaluation, including cerumen removal, and were thereafter rescreened. Rescreening took place within 2 weeks of the referral for management.

Each child was screened bilaterally with the left ear tested first, using automated screening methods at frequencies from 500 Hz to 4000 Hz. The pass criterion was set at 25 dBHL (American Academy of Audiology, 2011). Other similar studies using automated technology used similar or higher pass criteria (Kam et al., 2014; Mahomed-Asmail et al., 2016b). During peak noise level, testing was interrupted until the noise levels reached below 50 dBA, again in accordance with the American Speech and Hearing Association (1997) and American Academy of Audiology (2011). If a child failed to respond to the required intensity level at one or more frequencies, he or she was rescreened. The rescreen followed the same process as the initial screen. The rescreen was conducted after a rest period was given. All screening results were saved to a password-protected folder and emailed to the audiologist for later review, as well as to ensure proper record keeping of screening results.

#### Diagnostic phase

The qualified audiologist conducted on-site diagnostic measures using automated audiometry.

To test concordance, the agreement between screening and diagnostic tests, all participants underwent a diagnostic audiology assessment, irrespective of whether they passed the otoscopic examination and screening. Diagnostic testing was performed on-site by an audiologist (SG) after the participant was set up and instructed by the facilitator. Automated audiometry was used to conduct the diagnostic phase of testing. All participants were given a rest period of an hour or longer between screening and diagnostic testing. Six grades of hearing loss, from slight to profound, were classified according to Clark (1981). The clinical protocol utilised for both screening and diagnostic testing is presented in Figure 1.

All data were saved to a password-protected folder with only the researchers having access. Data were entered and verified by both researchers. All forms and audiograms were stored in a locked cabinet after data entry was complete.

### Data analysis

Frequency distributions were calculated for all screening and diagnostic tests. Sensitivity (%) and specificity (%) scores as well as the positive and negative predictive values (PPV/NPV) were calculated using two-by-two contingency tables. Sensitivity reflects the probability of the automated test being positive when the hearing loss is truly present. Specificity indicated the probability of the automated test being negative when the hearing loss was truly absent. PPV represents the percentage of participants with a positive result who actually have hearing loss, whilst NPV reflects the percentage of participants with a negative test outcome who truly do not present with hearing loss.

## Ethical consideration

Ethics approval was obtained from the Biomedical Research and Ethics Committee of the University of KwaZulu-Natal (certification number: BE288/15). Ethics approval was also obtained from the Department of Basic Education within the North West province. Participants received information letters, consent forms and letters of assent. Participants were informed of their right to withdraw at any stage of the study. Participants who required management were appropriately referred, and all participants received a copy of their results to take home to their parents. Data were entered onto a password-protected computer, and names were replaced with research numbers to ensure anonymity and confidentiality.

## Results

A total of 146 ears were assessed. For those participants who were rescreened at one or more frequencies, only the rescreen results were used for analysis.

### Otoscopic examination

Based on otoscopic findings, four ears (3%) required referral for cerumen management, three for partial impaction of wax and one for complete occlusion. They were referred for cerumen removal then rescreened. These participants also underwent diagnostic audiometric testing.

### Automated screening and diagnostic outcomes

In all, 15 ears (10.3%) of 13 participants failed the automated screening test. A total of 16 participants were rescreened after obtaining a fail result on the initial screen and 13 participants obtained a fail result on the rescreen, whilst 3 participants passed the rescreen and were given an overall pass result. For diagnostic testing, 23 ears (15.7%) of 20 children were identified as having a hearing deficit. Eight ears that passed the screening test result failed the diagnostic test. Therefore, there were eight false-negative screening tests but no false-positive tests. The sensitivity, specificity, PPV, NPV and diagnostic accuracy of the automated test are shown in Table 1. The sensitivity was low (65.2%) indicating that a total of eight ears that passed the screening test failed the diagnostic assessment. The specificity was high (100%), which implied that all participants who truly did not have hearing loss were correctly identified by the automated hearing assessment.

**TABLE 1:** Outcome of screening and diagnostic tests.

Variable	Diagnostic test positive	Diagnostic test negative	%	95% confidence intervals
Screening test (positive)	15	0	-	-
Screening test (negative)	8	123	-	-
Sensitivity	-	-	65.2	42.77% – 83.61%
Specificity	-	-	100	97.04% – 100%
Positive predictive value	-	-	100	78.19% – 100%
Negative predictive value	-	-	93.9	88.31% – 96.42%
Diagnostic accuracy	-	-	94.5	89.7% – 97.33%
Disease prevalence	-	-	15.7	10.26% – 22.69%

All hearing losses identified were sensory-neural in nature. Mild hearing loss was identified in 11 ears (8%), moderate in 7 (5%), severe in 3 (2%) and profound in 2 ears (1%). Seventeen ears (12%) presented with unilateral hearing impairment and six ears of three participants (4%) had bilateral hearing impairment.

The eight false-negative screening test results were further analysed (Table 2). Three ears showed low-frequency sensory-neural hearing loss, whilst five ears showed a low- to mid-frequency hearing loss, which ranged from mild to moderate.

**TABLE 2:** Thresholds across frequency ranges for diagnostic (D) and screening (S) tests for eight false-negative screening tests.

Number	125 Hz	250 Hz	500 Hz		1000 Hz		2000 Hz		4000 Hz		8000 Hz
	D	D	D	S	D	S	D	S	D	S	D
1	**30**	**30**	**40**	**25**	25	25	25	25	25	25	25
2	**35**	**40**	**35**	**25**	**35**	**25**	15	25	20	25	20
3	**65**	**50**	**60**	**25**	**30**	**25**	**30**	**25**	**50**	**25**	**45**
4	**60**	**60**	**35**	**25**	**35**	**25**	20	25	10	25	5
5	**60**	**65**	**40**	**25**	**30**	**25**	15	25	10	25	10
6	**30**	**35**	**35**	**25**	**40**	**25**	25	25	25	25	15
7	**55**	**60**	**30**	**25**	25	25	20	25	15	25	15
8	**35**	**40**	**35**	**25**	25	25	25	25	10	25	10

Note: Discrepancies between the tests are in bold.

## Discussion

This study showed the results of a combination of asynchronous telehealth and automated testing methods to deliver both screening and diagnostic hearing services in a rural school context. Diagnostic testing performed at the school overcomes the risk of loss to follow-up for diagnostic testing that occurs in conventional screening services. The present study had a high specificity (100%) but a lower sensitivity (65.2%). This is similar to the findings of Cardoso et al. (2014) where the sensitivity and specificity of a portable hearing screening device were determined. They felt that modification of screening level criteria may be necessary when working with different population groups. When using a new device, especially automated devices, audiologists should carefully evaluate the protocols proposed for the device and if necessary conduct trial studies that specifically evaluate modifications to the test protocols. The KUDUwave, for example, suggests a response time of 1500 m/s. This time may be too short for children to respond and as a result this may be recorded as a no response. Such protocol issues can be adjusted through future studies investigating response time and its impact on test performance.

It is understood no screening measure is likely to identify all true positives and true negatives (Stein, 1999), because of the nature of hearing screening measures where pass and fail criteria are based on a predetermined intensity level. However, it is important that the screening outcomes reflect a low false-positive rate (high specificity), and more importantly a low false-negative rate (high sensitivity) (Clemens & Davis, 2001). Fletcher, Fletcher and Wagner (1996) emphasised that screening tests are most useful to the clinician if they have a high sensitivity rate. An evidence-based literature review of eight studies for pure-tone screening tests for children reported sensitivities of 0.12% – 97% and specificities of 0.5% – 99%. Definitions of hearing loss, response criteria and technologies used varied between the eight studies reported (Prieve, Schooling, Venediktov, & Franceschini, 2015).

The false-negative tests in this study are of concern. Whilst the authors could not find a definitive explanation, possible reasons for the false-negative tests could relate to the device, the testing procedure or child-related variables. Distractibility of some of the participants during diagnostic testing was a concern, especially the younger ones (6-year olds). Because of this, they may have not been focused during diagnostic testing, therefore failing the test. The diagnostic and screening tests used the same device, so tone generation and volume should have been consistent. The KUDUwave device has been validated for diagnostic testing in several studies (Brennan-Jones et al., 2017; Visagie, Swanepoel, & Eikelboom, 2015). What may have differed in the two test situations might have been the depth of insertion of the foam ear tips. The facilitator inserted them for both screening and diagnostic testing, and it is speculated that they might not have been inserted as deeply for diagnostic testing as they were during screening. Ambient noise has been cited as a reason for failure to diagnose low-frequency hearing loss (LFHL). The same noise attenuation methods and headphones were used in all tests, both screening and diagnostic. Diagnostic testing took place an hour after screening and the noise levels may have risen and exceeded the noise-dampening capacity of the Ambidome headphones and insert earphones. When the ambient noise level was routinely checked, it was mostly lower than 50 dBSPL during testing. Based on the environmental noise control measures undertaken, which is slight as well as on the noise control features of the device, it is unlikely that noise could have significantly impacted on the test result. Another possible reason could be related to the lack of counterbalanced testing for this study. The same order of testing was used for all children and this may have resulted in a learning effect for listening.

Three of the false-negative screening tests in this study presented with an LFHL and five of the eight false-negative tests failed to identify hearing loss at 1000 Hz. Low-frequency hearing loss is prevalent in this age group and is consistent with other studies (Olusanya, Neuman, & Saunders, 2014; Rao, Subramanyam, Nair, & Rajashekhar, 2002), which report a prevalence of mild hearing loss in school-aged children of around 11.3%, which is slightly higher than the prevalence of 8% found in the present study. Mild hearing loss is not easily identified and as a result is most often underestimated in terms of its impact on academic development (Dodd-Murphy, Murphy, & Bess, 2014).

Hyde (2016) mentions that no screening programme could achieve perfect sensitivity and that ‘real-world’ screening tests are likely to miss some patients. This point may inevitably outline the benefits of using telehealth-based audiology service delivery models in schools, as screening and subsequent diagnostic testing could be conducted immediately after screening.

Other studies using automated audiology technology such as Automated Auditory Brainstem Response testing and Otoacoustic Emissions yielded a small percentage (<20%) of false-negative results (Hyde, Riko, & Malizia, 2006; Schmidt, Sataloff, Newman, Spiegel, & Myers, 2001) and protocol revisions, noise control measures and equipment calibration were some measures mentioned applied to improve test sensitivity. However, it is important to note that the above mentioned tests are objective measures, therefore not requiring a response from the patient. It is plausible to assume that with subjective measures such as in the case of pure-tone audiometry, human-related variables such as distractibility and fatigue could also add to reduced test sensitivity rates.

Careful protocol consideration is imperative when conducting a hearing evaluation programme. Although not investigated in the study, the extension of the response time on the automated software provided more time for the learner to respond. This is especially necessary when evaluating children because of their uncertainty and fluctuating attention levels. Screening protocols should also carefully consider their criteria in terms of screening intensity levels. The majority of the screening protocols recommend that screening be conducted at 20 dBHL (SASLHA, 2011); however because of the high-noise environments, screening intensity levels have often been raised to 25 dBHL, or even to 30 dBHL, in order to avoid false-positive referrals (Mahomed-Asmail et al., 2016a, 2016b). An intensity level of 25 dBHL was used to sensitise the test battery to identify mild hearing losses (Dodd-Murphy et al., 2014). Several studies highlight the concern of mild hearing loss among participants (Olusanya et al., 2014; Samelli, Rabelo, & Vespasiano, 2011). The protocol was also successful in identifying unilateral hearing loss and this was most prevalent in the present study. Unilateral hearing loss is a concern in the school-aged population and can result in challenges with localisation and speech perception in background noise (Lieu, 2013).

The study found that children presented with primarily unilateral mild hearing impairment. This finding was consistent with other studies, which indicate that this type of hearing loss is prevalent in the school-aged population (Butler, 2012; Govender et al., 2016). Based on diagnostic testing, children were referred for management, including further diagnostic testing that included speech audiometry, as well as for a hearing aid evaluation.

This study confirmed that a facilitator can be trained to perform video-otoscopy and pure-tone screening using an automated audiometric device in a school during teaching hours. The results can be transmitted to an audiologist for review at a later time. Diagnostic testing can also be performed in the school using the same device. For this study diagnostic testing was undertaken by an audiologist on site, but it can also be performed remotely by an audiologist with the facilitator setting up the patient and being supervised in real-time over the Internet. Automated audiometry because of its predetermined protocol also allows for an adequately trained facilitator to execute the test under the remote observation of the audiologist. The results could then be forwarded to an audiologist for later review and management decisions. For the developing world, task shifting and enabling a facilitator to perform both screening and diagnostic testing on site in a school would be the most efficient use of scarce resources. Other similar studies also document successful outcomes when using a trained facilitator in an underserved context (Biagio, Swanepoel, Adeyemo, Hall, & Vinck, 2013). Future studies should focus on obtaining facilitator feedback regarding the process so that improvements can be made.

## Conclusion

The study investigated the outcomes of a telehealth-based audiology service model using automated audiometric testing to screen hearing and confirm hearing loss in children at a rural South African school. The findings revealed that automated audiometry used within an asynchronous model can identify hearing loss in school-aged children; however, the issue of the false-negative rate obtained in this study was of concern. It is therefore recommended that ongoing evaluation occurs for automated devices to improve their reliability and validity particularly in relation to child-related factors. When testing children, it is important to ensure that they are attentive and alert to avoid effects of distractibility on test results. Counterbalancing testing order especially with children is important so as to avoid a learning effect for listening, and this was a limitation of the study probably contributing to the high false-negative rate. These factors support the increasing need for practice guidelines to be developed for tele-audiology to ensure standardised practice. Future research should focus on developing other automated audiology tests and implementing these within the school context so that a comprehensive test battery of automated audiology tests can be provided within a telehealth model.
